# Evaluating the Immune Response in Rabbits to an Escalating Dose of mRNA-Based HIV-1 Env Immunogens

**DOI:** 10.3390/vaccines13111161

**Published:** 2025-11-14

**Authors:** Shamim Ahmed, Durgadevi Parthasarathy, Tashina C. Picard, Gary R. Matyas, Mangala Rao, Alon Herschhorn

**Affiliations:** 1 Division of Infectious Diseases and International Medicine, Department of Medicine, University of Minnesota, Minneapolis, MN 55455, USA; ahme0242@umn.edu (S.A.); parth053@umn.edu (D.P.);; 2 U.S. Military HIV Research Program, Center for Infectious Diseases Research, Walter Reed Army Institute of Research, 503 Robert Grant Avenue, Silver Spring, MD 20910, USA; gmatyas@hivresearch.org (G.R.M.); mrao@hivresearch.org (M.R.); 3 Institute for Molecular Virology, University of Minnesota, Minneapolis, MN 55455, USA; 4 Microbiology, Immunology, and Cancer Biology Graduate Program, The College of Veterinary Medicine Graduate Program, and the Molecular Pharmacology and Therapeutics Graduate Program, University of Minnesota, Minneapolis, MN 55455, USA

**Keywords:** HIV-1, vaccine, antibodies

## Abstract

Background: The development of an effective HIV-1 vaccine remains a major challenge due to HIV-1’s extraordinary diversity, high mutation rate, and the rarity of broadly neutralizing antibody (bnAb) precursors. To address these challenges, we have previously immunized rabbits with mRNA-LNPs encoding for HIV-1 envelope glycoproteins (Envs), together with mRNA-LNPs encoding for HIV-1 Gag, which likely mediated the generation of virus-like particles presenting HIV-1 Envs to the immune system in vivo. Methods: Here, we investigated whether an escalating dose (ED) immunization using mRNA-LNP priming, followed by boosts with synthetic, protein-based, virus-like particles (synVLPs) displaying HIV-1 SOSIP trimers via SpyTag/SpyCatcher conjugation (group 1), could improve the quality and durability of the antibody responses compared to conventional bolus immunization (group 2). Previous studies have shown that, in contrast to single bolus immunization, the ED priming strategy could enhance B cell activation and prolong affinity maturation, resulting in higher-quality antibody responses. Results: Upon vaccination, rabbits from both groups developed strong homologous anti-Env antibody responses, with an increasing ability of sera from immunized rabbits to bind Envs following subsequent boosts. Antibodies in rabbit sera bound heterologous Envs, but there was no statistically significant difference in binding between the two groups. Overall, antibody responses were comparable across all animals and declined similarly over time in both groups, indicating that neither the adjuvants nor the ED priming led to any marked differences within this small sample size. Neutralization activity against homologous tier-2 HIV-1_AD8_ (mRNA prime) and tier-2 HIV-1_1059_ (protein boost) was generally low across both groups; however, a higher neutralization titer was observed for the ED group against HIV-1_AD8_ following the final boost. One of the rabbits from the bolus group exhibited exceptionally high neutralization titers that correlated with elevated Env-specific binding against HIV-1_1059_. Conclusions: These results highlight the challenges in eliciting broad and potent neutralizing antibody (nAb) responses. Our findings underscore the need for the continued development and refinement of immunogen design and delivery strategies to guide the elicitation of nAb.

## 1. Introduction

As of 2024, approximately 40.8 million people are living with HIV-1 (PLWH) around the world [1]. Despite increasing knowledge of HIV-1 biology and pathogenesis over the years, there is still no cure or vaccine for HIV-1 infection. Substantial efforts have led to the development of new vaccine candidates and platforms with the aim of eliciting antibodies capable of neutralizing HIV-1 entry. Of all the efficacy trials tested to date, only one trial, RV144, showed a weak–moderate and temporal vaccine efficacy in humans, with approximately 31.2% temporal protection from HIV-1 infection [2,3,4,5,6,7,8,9,10,11,12,13,14]. bnAbs are developed only in a small subset of PLWH and after months or years of chronic infection and extensive somatic hypermutation [15,16,17,18,19]. Rapid mutation rate, alternative glycosylation patterns, and conformational flexibility of HIV-1 Envs [20,21,22] impose strict requirements for an effective vaccine that must elicit cross-reactive, broad, and potent protection against diverse viral variants [23,24]. In addition, HIV-1 can escape nAbs by spreading via cell–cell transmission [25,26]. After immunization, the germinal center (GC) typically plays a vital role in improving the breadth and affinity of developed antibodies through rounds of extensive selection against HIV-1 Envs [27,28]. Affinity maturation in GC can influence the pool of memory B cells and the generation of long-lived plasma cells that remain long after immunization [28,29]. Studies in rhesus macaques have shown that the size of the early GC response is linked to the strength of nAb responses [30]. Thus, enrichment of B cell clones in the GC may be beneficial for a response against difficult-to-neutralize pathogens like HIV-1, as it increases the diversity of B cells, some of which may contain germline antibody sequences that could develop into affinity-matured bnAbs [31,32].

Several approaches have been used by different groups to study and mimic the molecular events that could trigger an effective antibody response by vaccination. These include immunogen design and the route and timing of vaccine administration to improve the quality and durability of elicited antibodies. Notably, some vaccine candidates may show greater efficacy when delivered through mucosal routes, as opposed to systemic routes [19]. Furthermore, the efficacy of vaccines may be impacted by the specific timeline of administration. Recent studies have demonstrated that subunit protein vaccines delivered over 7–14 days in an ED can enhance immune responses compared to traditional single-dose (bolus) immunizations [30,33,34]. Moreover, this strategy was associated with more durable germinal centers lasting up to 2 months [34], extensive antibody maturation, reduced immunodominance, and the generation of broader neutralizing responses.

**Figure 1:**
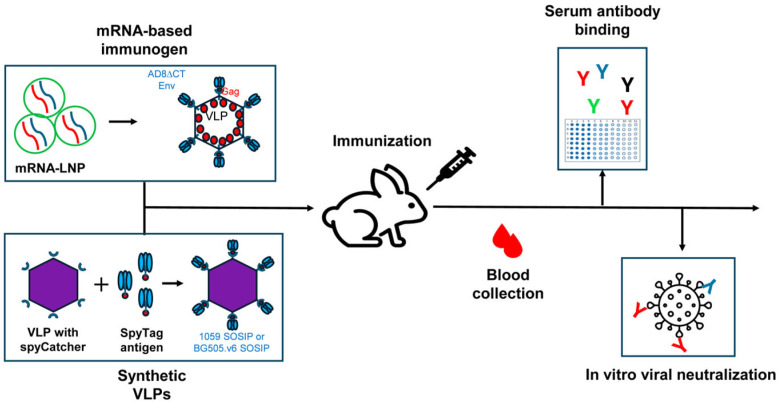
A scheme of immunization and evaluation of the immune response. Rabbits were immunized with both mRNA-LNP and synVLP presenting HIV-1 Envs. Blood was collected between immunizations, and sera or plasma were assessed for Env binding and evaluated for neutralization activity in vitro.

Although antibodies have been shown to protect against different infectious diseases, the addition of cellular immunity may be beneficial for the overall immune response. Pre-clinical studies on non-human primates (NHPs) have demonstrated that high titers (greater than 1:500) of vaccine-induced antibodies may be needed to protect against simian-human immunodeficiency virus (SHIV) rectal challenge [35]. Thus, the combination of both humoral and cellular arms (B and T cells) of the immune system may be beneficial for the prevention of HIV-1 acquisition [36]. In typical immunity settings, bnAbs target free viruses and can mediate antibody-dependent cellular cytotoxicity (ADCC), while other non-neutralizing antibodies can still mediate ADCC through Fc-mediated effector functions. T cell immunity can target and eliminate virus-infected cells [37,38]. Recently, a phase I clinical trial reported that mRNA-based vaccines encoding membrane-anchored HIV-1 envelope trimers could elicit strong B and T cell responses, with 80% of participants developing autologous tier-2 nAbs [39]. In contrast, among all the participants who received soluble trimer, only 4% elicited autologous tier-2 nAbs. Moreover, mRNA-based platforms allow co-expression of HIV-1 structural proteins (i.e., HIV-1 Gag) and HIV-1 Envs in the same cell, which can generate VLPs and present membrane-anchored HIV-1 Envs to the immune system [40,41,42] (Figure 1). Together, these considerations highlight the importance of optimizing both immunogen design and immunization strategy to enhance both humoral and cellular immunity. Building on these insights and our previously published rabbit immunization data [40], here we investigated the effects of an ED immunization strategy using mRNA-LNPs on the potency and breadth of the elicited antibodies.

We divided six rabbits into two groups of three rabbits each and primed them with an mRNA-LNP encoding HIV-1_AD8∆CT_ and HIV-1 Gag, followed by protein boosts. Rabbits in group 1 (ED group) were immunized with mRNA-LNPs, with the total amount distributed in an escalating manner over alternating days across 6 days (three doses in total), followed by a single mRNA-LNP boost at week 5. Rabbits in group 2 were immunized with similar mRNA-LNP amounts but using a conventional bolus approach; both groups received the same mRNA-LNP dosage (35 µg/rabbit) for the prime and first boost. All the rabbits were subsequently boosted with synVLPs displaying the 1059 and BG505.v6 SOSIPs, formulated with adjuvants. The synVLP boosts were formulated with adjuvants, whereas mRNA-LNPs did not include any adjuvants. Two different adjuvants were used in this study. Group 1 received the Army Liposome Formulation with QS-21 (ALFQ), a liposome-based adjuvant that has been shown in animal models to induce strong antigen-specific IgG1 responses along with balanced T helper 1 (Th1) and T helper 2 (Th2) immunity, as described previously for the Army Liposome Formulation (ALF) family of vaccine adjuvants [43]. Group 2 received AddaVax, a squalene-based oil-in-water emulsion that is similar to the commercially licensed adjuvant MF59, which has been used for influenza vaccines in Europe [44]. AddaVax has also been reported to elicit both Th1 and Th2 immune responses [45,46]. Our previous vaccine study identified the co-administration of mRNA-LNPs encoding for cytoplasmic-tail deleted (∆CT) Envs and LNPs encoding for HIV-1 Gag as the most effective immunogen combinations to elicit protective immunity, presumably by high-level presentation of HIV-1 Env on VLPs in vivo. Here, we utilized these effective immunogens in combination with different adjuvants under distinct immunization strategies to further study their effects on the immune response.

## 2. Materials and Methods

### 2.1. Rabbit Immunizations and Blood Collections

All experimental protocols involving animals were reviewed and approved by the Institutional Animal Care and Use Committee (IACUC; protocol# 2409-42436A) of the University of Minnesota. Six female New Zealand White rabbits at 6 months old were housed according to National Institute of Health (NIH) guidelines for housing and care of laboratory animals. Rabbits were housed individually but in adjacent cages that allowed interaction, minimizing stress and promoting natural social behavior. Rabbits were intramuscularly immunized in the epaxial muscle with mRNA-LNPs every other day for the ED group (35 μg/rabbit) and with a single shot for the bolus group. Animals were subsequently boosted with a bolus shot of mRNA-LNP (35 μg/rabbit) and synVLP-1059 and BG505.v6 SOSIP (25 μg/rabbit). SynVLP immunogens were formulated with ALFQ adjuvant for group 1 and with AddaVax adjuvant (InvivoGen, San Diego, CA, USA) for group 2 in a 1:1 ratio. Pre-immune blood was collected before the first immunization and then subsequently before every boost. Blood samples were collected from the marginal ear vein or central ear artery following an approved IACUC protocol. All animals received an IV catheter in the ear vein, and blood was collected via catheter. Blood was collected from the marginal ear vein using a rabbit restraining device, with all procedures conducted by trained personnel to minimize animal stress and discomfort. Anesthesia was not used during the procedure. To facilitate blood flow, rabbits’ ears were gently rubbed with gauze soaked in wintergreen oil before collection.

### 2.2. Enzyme-Linked Immunosorbent Assay

To test serum binding to different trimers, we used an in-house Enzyme-Linked Immunosorbent Assay (ELISA) as previously described [40]. Briefly, 96-well Immulon 2HB plates (ThermoFisher, Waltham, MA, USA) were coated overnight at 4 °C with 0.1 µg/well antigen in 100 µL. The next day, plates were washed 3 times with 0.1% Tween 20 (Bio-Rad, San Francisco, CA, USA) in PBS (wash buffer) and blocked overnight at 4 °C with 0.5% dry skim milk in PBS containing 0.1% Tween 20 (blocking buffer). Plates were washed again 3 times the following day with wash buffer and incubated for 1 h at RT with rabbit plasma diluted in blocking buffer. After washing the plates 3 times with wash buffer, the wells were incubated with a 1:50,000 dilution of Peroxidase AffiniPure F(ab’)_2_ Fragment Goat Anti-Rabbit IgG (H + L) (cat # 111 036 045; Jackson ImmunoResearch, West Grove, PA, USA) for 1 h at RT. Next, the wells were washed 3 times, and 100 μL of TMB solution (300 µL of 4 mg/mL 3,3,5,5-tetramethyl-benzidine (Sigma, St. Louis, MO, USA) in DMSO, 9.7 mL of 0.1 M sodium acetate, pH 5.0, and 5 μL of fresh 30% hydrogen peroxide) was added to each well. After an incubation of 1 h at RT, the HRP reaction was stopped by adding 50 μL of 0.5 M H_2_SO_4_, and optical density was measured at 450 nm using a Synergy H1 spectrophotometer (Agilent, Santa Clara, CA, USA).

### 2.3. In Vitro Transcription, mRNA Purification and mRNA-LNP Preparation

The HIV-1_AD8_ Envs encoded by the mRNA were used with the CT deleted immediately after residue 708. mRNA for immunization was generated as previously described [40]. Briefly, in vitro transcription (IVT) plasmids consisting of a bacteriophage T7 RNA polymerase promoter, 5′ and 3′ untranslated regions, a gene of interest, and a polyA tail were linearized by incubating for 4 h at 37 °C with SapI restriction enzyme. The resulting linearized DNA was analyzed by agarose gel electrophoresis to confirm successful linearization and purified using the Wizard(R) SV Gel and PCR Clean-up System (Promega Madison, WI, USA, cat # A9282). Linearized DNA was then used as a template in the IVT assay using the T7-FlashScribe Transcription kit (Cellscript, Madison, WI, USA, cat # C-ASF3507) and CleanCap (TriLink Biotechnologies, cat # N-7113-10). We replaced uridine with pseudouridine (N1-Methyl-Pseudouridine-5′-Triphosphate, TriLink Biotechnologies, San Diego, CA, USA, cat # N-1081-10). At the end of incubation, DNase was added to the reaction, which was then incubated for another 15 min. The mRNA was then purified using the Megaclear kit (ThermoFisher (Invitrogen), Waltham, MA, USA, cat # AM1908). The concentration of the purified RNA was measured by NanoDrop. The mRNA was then incubated with cellulose (Sigma-Aldrich, cat # C6288), which was prewashed with 1× chromatography buffer (0.2 mg/mL) for 10 min while shaking (400 rpm) to remove dsRNA. The eluted mRNA was then transferred to each spin column and spun down to remove the buffer. The mRNA was then again purified with the Megaclear kit. The mRNA was diluted to a concentration of 1000 ng/µL and stored at −80 °C until encapsulated in lipid nanoparticles by Acuitas Therapeutics Inc. (Vancouver, British Columbia, Canada). Both Env and Gag mRNAs were co-formulated in a single LNP preparation in a 1:1 molar ratio.

### 2.4. Protein Expression and Purification

Freestyle 293F cells (ThermoFisher (Gibco), Waltham, MA, USA, cat # 11625019) were transiently transfected at a density of 2 × 10^6^ cells/mL with a SOSIP- or SpyTag SOSIP-expressing plasmid and a human furin-expressing plasmid at a ratio of 4:1 using Turbo293 transfection reagent (Speed Biosystems; Gaithersburg, MD, USA). Post-transfection, transfected cells were then incubated for 5–7 days on a shaker in a tissue culture incubator at 37 °C, 8% CO_2_. After incubation, supernatants were clarified by centrifugation at 10,000× *g* for 30 min and filtered through a 0.2 μm filter (VWR). Supernatants were loaded on a *Galanthus nivalis* lectin (GNL) column (Vector Laboratories) at 4–8 °C. The column was equilibrated with PBS before loading the supernatant. After running the sample, the column was washed with PBS, and the protein was eluted with 1 M Methyl-α-D-mannopyranoside/PBS solution, filtered through a 0.2 μm filter, and concentrated using Vivaspin 6 centrifugal concentrators (30 kDa; Cytiva, Marlborough, MA, USA). Purified SOSIP Envs were then separated using a SEC column HiLoad 16/600 Superdex 200 (Cytiva). The trimer fractions were pooled, concentrated, and stored in aliquots at −80 °C until use.

### 2.5. SOSIP Display on Synthetic Viral-like Particles

The 1059 and BG505.v6 SOSIP-stabilized Envs used on the synVLPs were obtained from previously published constructs containing the A501C and T605C (“SOS”) and I559P (“IP”) mutations [47]. The membrane proximal external region (MPER), transmembrane (TM), and CT regions were removed to make it soluble. The REKR sequences were replaced with R6 to enhance cleavage, and the native signal peptide was substituted with the human tissue plasminogen activator (TPA) signal sequence. Purification of 1059-SOSIP-SpyTag and SpyCatcher nanocages was previously described [40,48]. Briefly, SOSIP Envs expressing C-terminus SpyTag were incubated with synVLPs expressing SpyCatcher at different ratios (between 1:1 and 6:1) in 25 mM Tris–HCl, 150 mM NaCl, pH 8.0, at 4 °C for 16 h. After incubation, aggregates were removed by centrifugation at 16,900× *g* for 30 mins at 4 °C. Conjugated proteins were separated on an 8–16% Mini PROTEAN TGX stain-free gradient gel (Bio-Rad) and imaged using a ChemiDoc XRS+ imager (Bio-Rad, San Francisco, CA, USA) to evaluate the optimal ratio for conjugation, which was then used for large volume preparation. Unconjugated trimers were removed by size exclusion chromatography using a HiLoad 16/600 Superdex 200 pg column (Cytiva, Marlborough, MA, USA). SynVLPs displaying SpyCatcher on their surface were expressed and purified following protocols adapted from Rahikainen et al. [48].

### 2.6. Rabbit Anesthesia and Euthanasia

Rabbits were humanely euthanized by trained personnel at the University of Minnesota animal facility under the supervision of a licensed veterinarian and in compliance with the approved IACUC protocol. Animals were sedated with an intramuscular (IM) cocktail consisting of ketamine (35 mg/kg), xylazine (5 mg/kg), and acepromazine (1 mg/kg), administered into the epaxial muscles. Due to large injection volume, the dose was divided into two equal portions, with half delivered to the right epaxial muscle and half to the left. Once an adequate level of sedation was achieved, animals received 1 mL of euthanasia solution via intravenous (IV) catheter. Death was confirmed by cardiac auscultation.

### 2.7. Generation of Single-Round Pseudoviruses

Pseudoviruses were generated following the methods described in previous studies [49,50,51]. In brief, 293T cells were co-transfected with three plasmids: the packaging construct (psPAX2), a luciferase reporter plasmid (pHIVec2.luc), and an Env-expression vector using Effectene transfection reagent (Qiagen) or calcium phosphate. After a 48-h incubation, culture supernatants were harvested by centrifugation at 600–900× *g* for 5 min at 4 °C. The virus-containing supernatant was stored in single-use aliquots at −80 °C. We evaluated viral particles by measuring HIV-1 p24 antigen concentration in pseudovirus preparations using a commercial ELISA kit (cat # XB-1000, XpressBio, Frederick, MD, USA).

### 2.8. Viral Neutralization Assay

Neutralization activity was evaluated using a single-round infection of TZM-bl reporter cells (NIH AIDS Reagent Program) in 96-well white plates (Greiner Bio-One, Monroe, NC, USA, NC, USA) following established protocols [49,52]. Briefly, 30 μL of diluted plasma was mixed with 30 μL of pseudoviruses and incubated for 1 h at 37 °C in 5% CO_2_. Subsequently, approximately 7000 TZM-bl cells in 30 μL of DMEM were added to each well and cultured for 48 h. Cells were then lysed, and luciferase activity was measured with a 2-s integration time using a Centro XS^3^ LB 960 luminometer (Berthold Technologies, Baden Württemberg, Germany). The measured relative light units (RLUs) were normalized to the effect of the same serum dilution of pre-immune serum for each specific rabbit. Dose-response curves were generated, and inhibitory dilution 50 (ID_50_) was calculated by nonlinear regression using the logistic equation after it was imported into Prism 9. Inhibitory activity was expressed as the serum dilution leading to a 50% reduction in signal (ID_50_). The minimum serum dilutions tested were 1:25 (experiments 1 and 2). For samples with weak neutralization, ID_50_ values below the detection limit were extrapolated from the fitted curves, as lower dilutions could not be evaluated due to non-specific effects.

### 2.9. Statistical Analysis

All statistical analyses were performed using GraphPad Prism 9 (GraphPad Software, Boston, MA, USA). Plasma antibody binding and in vitro neutralization dose–response curves were fitted to the 4-parameter (logistic) equation after the model was imported, using non-linear regression in Prism 9. For analysis, the dose values were expressed as plasma dilutions rather than absolute concentrations, and the resulting parameter was reported as the half-maximal inhibitory dilution (ID_50_). The lowest dilution tested for plasma binding was 1:30, and for in vitro neutralization 1:25.

## 3. Results

**Figure 2:**
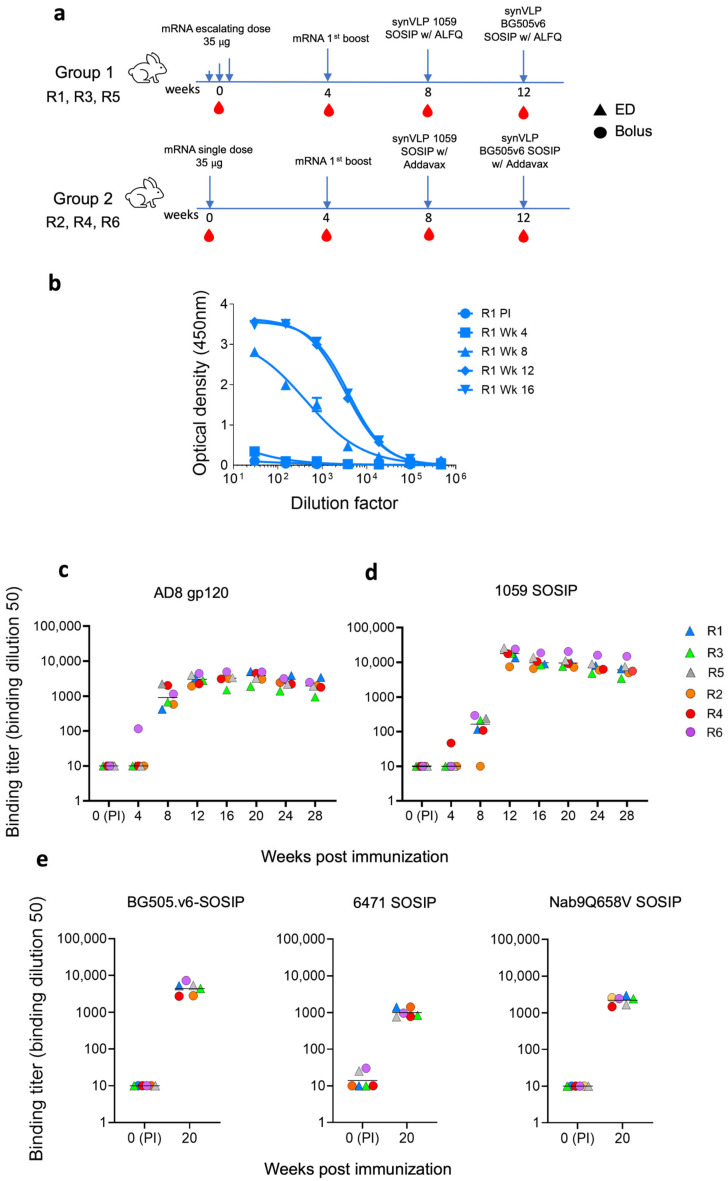
Induction of strong, high-titer binding antibodies by different immunization strategies. (**a**) An immunization scheme of different vaccination strategies for each group of rabbits: ED group (R1, R3, and R5) and bolus group (R2, R4, and R6). (**b**) Binding activity of IgG-containing sera from rabbit 1 (Rb1) to soluble HIV-1_AD8_ gp120 Env, measured by ELISA at the indicated time points. (**c**–**e**) Binding titers of sera from all six rabbits to HIV-1_AD8_ gp120 (**c**), HIV-1_1059_ SOSIP (**d**), and a panel of homologous and heterologous SOSIP Envs (**e**). We assessed antibody durability over time by monitoring antibody titers at weeks 20, 24, and 28 post-vaccination. Sera were collected at multiple time points during and post-immunization to evaluate antibody persistence. Since NAB9 SOSIP was poorly expressed, we instead used the NAB9Q658V mutant containing a trimer-stabilizing mutation [53]. Antibody binding titers varied over time, demonstrating both durability and gradual decline of the responses. For sera in which all dilutions resulted in optical density <0.12 or extrapolated ID50 values were <10, the ID50 values were set to 10.

We divided six rabbits into two groups (n = 3). Rabbits in group 1 were immunized with an ED of mRNA-LNPs, and rabbits in group 2 were immunized with an identical amount of immunogen but as a single administration dose (bolus immunization; Figure 2a). Rabbits were subsequently boosted with the same mRNA-LNP and synVLPs presenting different HIV-1 Envs to increase immunogen diversity presented to the immune system. Protein immunogens were formulated with two different adjuvants, ALFQ and Addavax, by mixing them at a 1:1 ratio with the synVLPs. We collected blood every 4 weeks, before each immunization, and tested the plasma for antibody binding using an in-house ELISA. We generated dose–response binding curves to soluble HIV-1 Envs at the indicated time points post-immunization (e.g., Figure 2b). Sera of all rabbits showed strong binding to gp120 over the course of immunization, with a titer greater than 1:3000 against the autologous AD8 gp120 at week 16 (Figure 2c). The median titer of all sera increased by 109-fold after the 1059 synVLP boost (median ID_50_ 1:163.6 vs. 1:17,901) (Figure 2d). Sera of all six rabbits contained strong binding antibodies to autologous BG505.v6 SOSIP after the BG505.v6 synVLP boost, with a median titer that was greater than 1:4800 (Figure 2e). Elicited antibodies exhibited broad binding according to sera binding to the heterologous 6471 SOSIP and NAB9_Q658V_ SOSIPs at week 20, with a median titer of 1:898 and 1:2427, respectively (Figure 2e). Antibody binding was subsequently assessed in weeks 20, 24, and 27, and all the rabbits maintained high-titer antibody binding throughout the study against autologous strains (Figure 2c,d). However, the binding titers gradually declined over time, suggesting a waning of antibody response in the later weeks post-boost.

**Figure 3:**
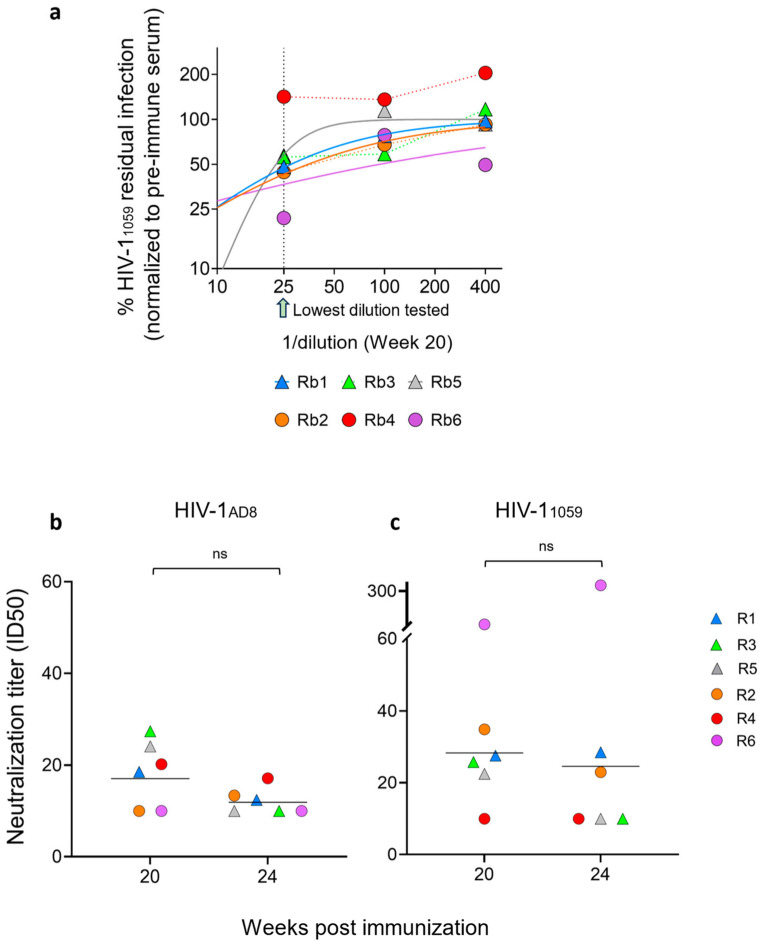
Sensitivity of HIV-1_1059_ and HIV-1_AD8_ Env pseudoviruses to rabbit sera. (**a**) Dilution response curves for the sensitivity of HIV-1_1059_ pseudoviruses to sera of all six rabbits. The dashed line represents the effect of the lowest serum dilution tested (1:25). The area below 25 (1:25) represents extrapolated curves. In cases of very weak responses, ID_50_ values were derived from curve fitting rather than direct experimental testing. (**b**,**c**) Neutralization titers (ID_50_) of sera collected at weeks 20 and 24 post-immunization against viruses pseudotyped with HIV-1_AD8_ (**b**) or HIV-1_1059_ Envs (**c**). Values below the detection threshold (ID_50_ < 10) were assigned a value of 10. The lowest serum dilution tested (1:25) and extrapolated ID_50_ values below this threshold are indicated. Neutralization data are representative of two independent experiments, each performed in duplicate. Data are shown as mean ± SEM with individual data points or as geometric mean titers.

To evaluate the effect of different immunization strategies combined with distinct adjuvants on the neutralization activity of elicited antibodies, we measured the neutralizing activity of rabbit sera collected at weeks 20 and 24 against HIV-1_AD8_ and HIV-1_1059_ using a single-cycle pseudovirus neutralization assay (Figure 3a). Overall, the rabbits exhibited no or weak neutralization activity against HIV-1_AD8_ pseudoviruses (Figure 3b). On week 20, sera of rabbits from the ED group (R1, R3, and R5) and of rabbit 4 from the bolus group (R2, R4, and R6) exhibited higher ID_50_ values against HIV-1_AD8_ pseudoviruses compared to the sera of rabbits 2 and 6 from the bolus immunization group. By week 24, ID_50_ values had decreased across both groups, and there was no difference between the groups. Similarly, sera of some rabbits from both groups demonstrated weak neutralization activity against HIV-1_1059_ at weeks 20 and 24 (Figure 3c). Interestingly, rabbit 6 from the bolus immunization group demonstrated exceptionally high neutralization titers at both time points (ID_50_ of 1:93 at week 20 and 1:335 at week 24; Figure 3). This robust neutralization activity strongly correlated with elevated antibody binding titers to 1059 SOSIP, which were substantially higher than those of other animals in the cohort (Figure 2). Except for rabbit 6, none of the sera effectively neutralized HIV-1_1059_ entry, underscoring the limited breadth of neutralizing responses in this study. In all neutralization assays, pre-immune sera from each rabbit were tested in parallel at equivalent dilutions to control for non-specific serum effects. Most serum dose–response curves could be reliably fitted to a logistic equation, while in cases of very weak responses, ID_50_ values below the 1:25 dilution threshold were extrapolated, as further testing was precluded by non-specific serum interference.

## 4. Discussion

The mRNA vaccine platform offers several advantages over traditional subunit vaccines, as it leverages the host translational machinery to generate the desired antigen anchored in the membrane of the cell surface. Moreover, mRNA-LNP technology allows encapsulation of multiple RNAs encoding different proteins (or co-administration of mRNA-LNPs encoding different proteins), which can mediate the delivery/formation of more complex immunogens (i.e., gag-mediated VLPs presenting surface antigen). Env trimers anchored on membranes may allow masking of non-neutralizing base epitopes and possibly direct antibody responses to more immunogenic sites [39]. Additionally, slow, escalated priming can markedly enhance antigen retention in germinal centers, allowing more time for B cells to undergo extensive affinity maturation and improve breadth [34]. In this study, we combined the advantages of mRNA-LNP technology, which enables membrane-anchored Env presentation, with a slow ED priming strategy designed to enhance antigen retention in germinal centers and drive extensive affinity maturation. However, continuous priming over a span of 2 weeks would seem impractical for a mass vaccination campaign, and we therefore modified our ED approach, which extends over a period of 6 days (three immunizations on alternate days). In our previous study, we showed that sequential immunization with conformationally diverse Envs yields low titer but broad neutralization activity in a subset of rabbits. Based on our findings, we utilized the same immunogens to study different immunization strategies in our current study. We compared the immunogenicity of ED versus bolus immunization strategies in rabbits using mRNA-LNP priming followed by synVLP boosts, combined with distinct adjuvants. Our findings highlight key insights into the durability and quality of antibody responses elicited by these vaccine regimens. We show that mRNA-LNP priming followed by synVLP boosts elicits strong binding antibody responses across all rabbits, with titers rising sharply after heterologous boosts and extending to heterologous Env variants. Over the period of subsequent assessment of plasma IgG binding, no significant differences were observed between the two groups (Supplementary Figures S1 and S2). Weak neutralizing activity was observed against HIV-1_AD8_ for all the rabbits, with a modest increase in neutralizing titer for the ED group compared to the bolus group, which diminished by week 24.

Neutralization activity against HIV-1_1059_ was also comparatively low, with no observable differences between the two groups. The notable exception was rabbit 6 from the bolus group, which developed unusually high neutralization against HIV-1_1059_, correlating with its exceptionally strong binding titers, suggesting that epitope-specific responses can drive functional neutralization in select cases. Given the small sample size (n = 3 per group), the observed differences should be interpreted as indicative trends rather than definitive outcomes. These findings highlight patterns worth following up in future studies with larger animal groups. Major limitations to our study are the limited sample size in each group and low neutralization activity. We previously showed broad, albeit low, neutralization activity in rabbits that were immunized with the same immunogen, and our current study expands that approach to improve antibody responses through different immunization strategies. Our study also did not include two additional groups: an ED group receiving Addavax and a bolus group receiving ALFQ, to allow comparison of the specific adjuvants for each mRNA-LNP dosing scheme. The rationale behind using ALFQ with the ED group was to improve the overall immune response to immunization based on previous studies. We used the Addavax adjuvant [54] in the bolus group as a reference based on our previous study [40].

## 5. Conclusions

Overall, these findings not only underscore the capacity of this approach to generate durable binding antibodies but also highlight the persistent challenge of eliciting broad and potent nAbs, emphasizing the need for further vaccine development, refined immunogen design, and improved immunization strategies.

## Data Availability

All data are available in the article and Supplementary Files.

## Supplementary Materials

The following supporting information can be downloaded at: https://www.mdpi.com/article/10.3390/vaccines13111161/s1.
