# Supplementary material for: Evaluating the Immune Response in Rabbits to an Escalating Dose of mRNA-Based HIV-1 Env Immunogens
Source: Vaccines (Basel). 2025 Nov 14;13(11):1161. doi: 10.3390/vaccines13111161 (PMC12656593; doi:10.3390/vaccines13111161)
Supplement: Supplementary file 1 [file vaccines-13-01161-s001.zip › vaccines-3951685-supplementary.pdf]

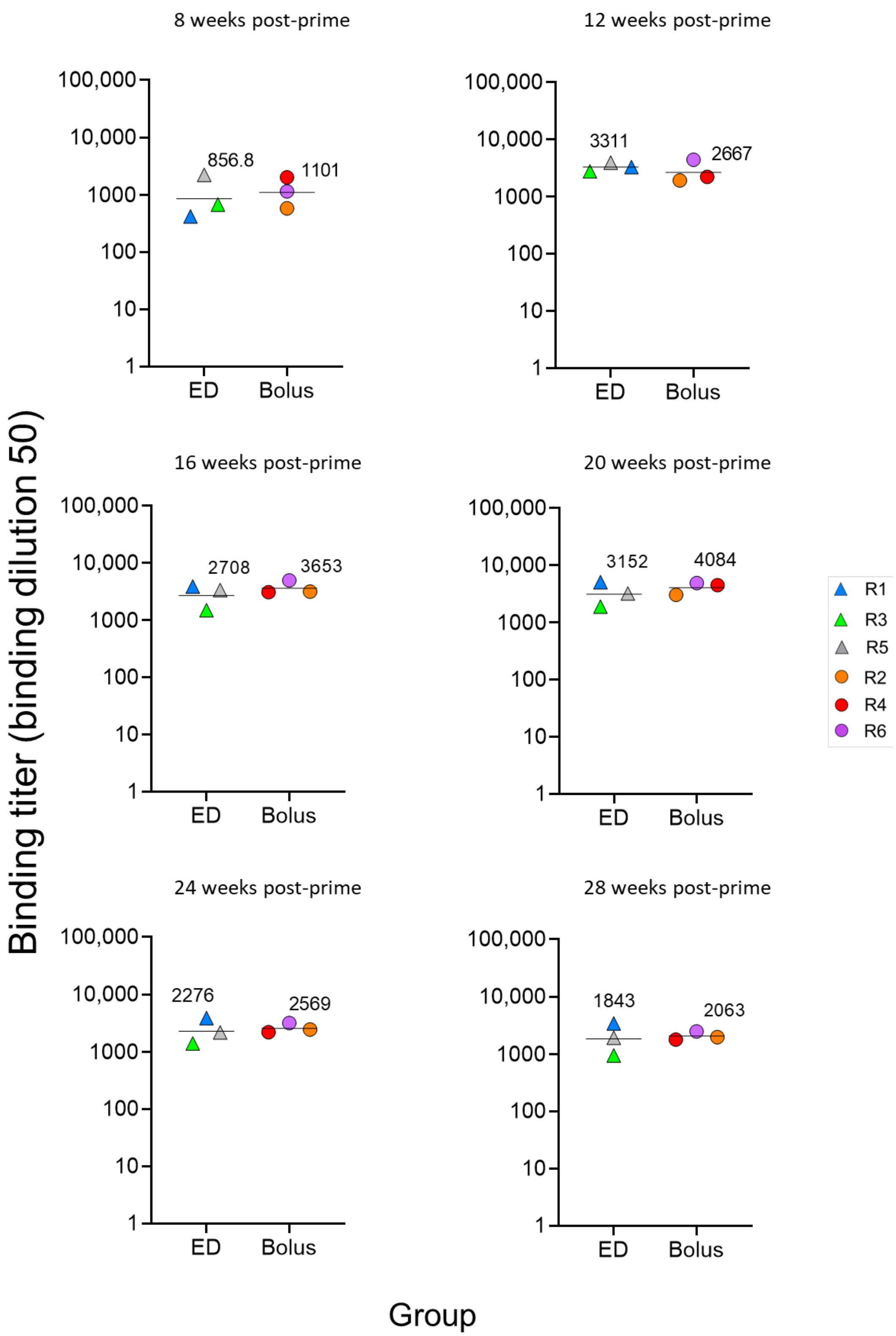

Supplementary Figure S1: **Comparison of antibody binding to HIV-1 $_{AD8}$ gp120 in the plasma of immunized rabbits from the two distinct immunization groups.** The geometric mean for each group in each specified week is shown in each graph.

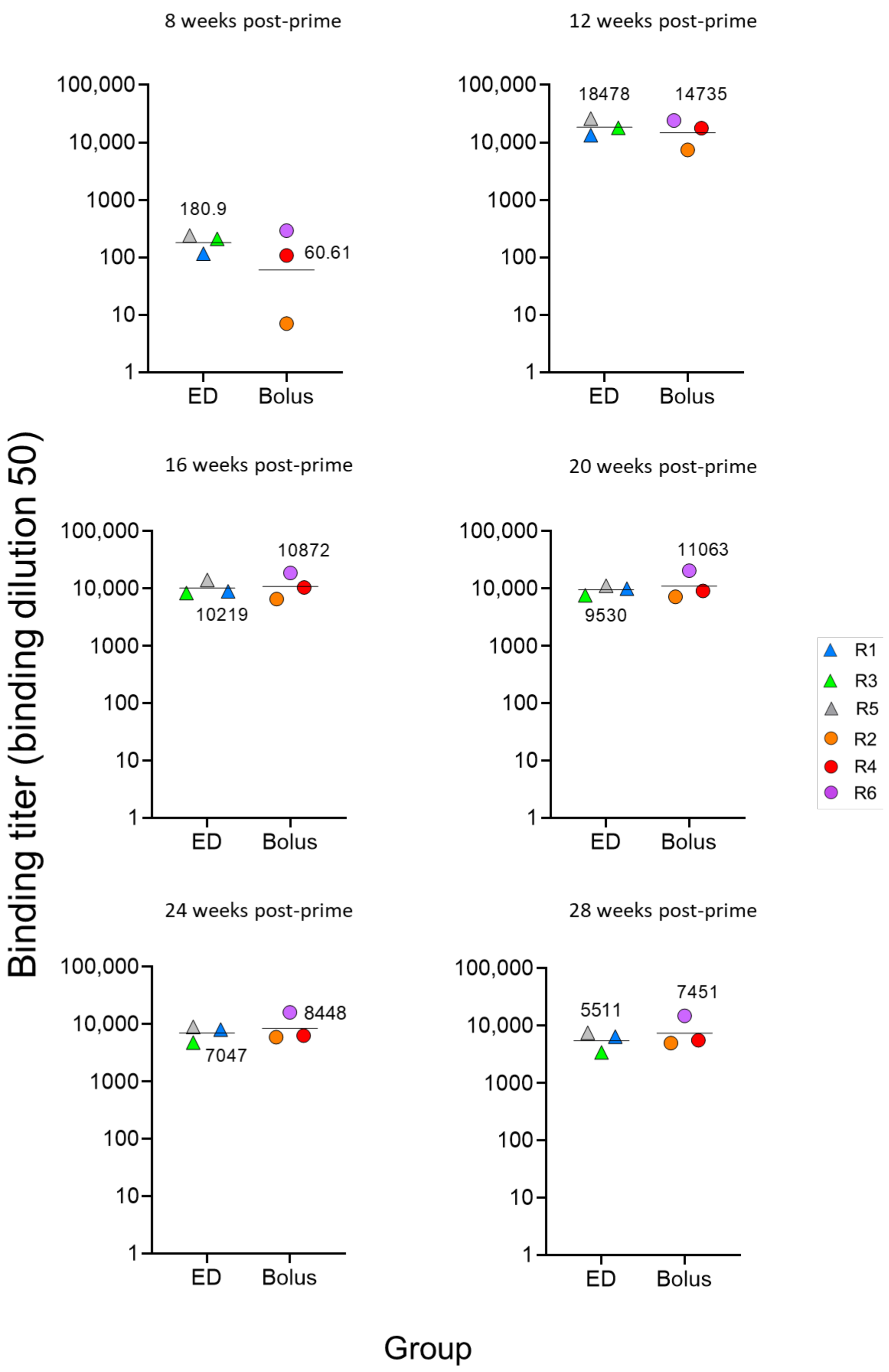

Supplementary Figure S2: Comparison of antibody binding to HIV-1$_{1059}$ SOSIP in the plasma of immunized rabbits from the two distinct immunization groups. The geometric mean for each group in each specified week is shown in each graph.
